# Tear Samples for Protein Extraction: Comparative Analysis of Schirmer's Test Strip and Microcapillary Tube Methods

**DOI:** 10.7759/cureus.50972

**Published:** 2023-12-22

**Authors:** May Ling Tham, Aidalina Mahmud, Maha Abdullah, Rafidah Md Saleh, Amirah Mohammad Razali, Yoke Kqueen Cheah, Niazlin Mohd Taib, Kok Lian Ho, Mazaya Mahmud, Muhammad Mohd Isa

**Affiliations:** 1 Department of Ophthalmology, Faculty of Medicine and Health Sciences, Universiti Putra Malaysia, Serdang, MYS; 2 Department of Community Health, Faculty of Medicine and Health Sciences, Universiti Putra Malaysia, Serdang, MYS; 3 Department of Pathology, Faculty of Medicine and Health Sciences, Universiti Putra Malaysia, Serdang, MYS; 4 Department of Biomedical Sciences, Faculty of Medicine and Health Sciences, Universiti Putra Malaysia, Serdang, MYS; 5 Department of Medical Microbiology and Parasitology, Faculty of Medicine and Health Sciences, Universiti Putra Malaysia, Serdang, MYS

**Keywords:** sodium dodecyl sulphate-polyacrylamide gel electrophoresis (sds-page), protein quantitation, tear sampling, microcapillary tube, schirmer’s test strip

## Abstract

Introduction: Tear sampling is an attractive option for collecting biological samples in ophthalmology clinics, as it offers a non-invasive alternative to other invasive techniques. However, there are many tear sampling methods still in consideration. This study explores the suitability of Schirmer’s test strip and microcapillary tube as reliable and satisfactory methods for tear sampling.

Methods: Tear samples were collected from eight healthy volunteers using the standard Schirmer's test strip method with or without anesthesia and microcapillary tubes. The total tear protein concentrations were analyzed via spectrophotometry and bicinchoninic acid (BCA) protein assay. The protein profile was determined by sodium dodecyl sulphate-polyacrylamide gel electrophoresis (SDS-PAGE). The optimal wetting length of Schirmer's strip and suitable buffer solutions were compared. Discomfort levels reported by participants and the ease of execution for ophthalmologists were also evaluated.

Results: Tear samples exhibited typical protein profiles as shown by SDS-PAGE. The mean total protein obtained from an optimum wetting length of 20 mm using Schirmer's strip without anesthesia in phosphate-buffered saline (PBS) yielded substantial quantities of protein as measured by nanophotometer (220.20 ± 67.43 µg) and the BCA protein assay (210.34 ± 59.46 µg). This method collected a significantly higher quantity of protein compared to the microcapillary tube method (p=0.004) which was much more difficult to standardize. The clinician found it harder to utilize microcapillary tubes, while participants experienced higher insecurity and less discomfort with the microcapillary tube method. PBS used during the tear protein extraction process eluted higher tear protein concentration than ammonium bicarbonate, although the difference was not statistically significant. Using anaesthesia did not ease the sampling procedure substantially and protein quantity was maintained.

Conclusion: Good quality and quantity of protein from tear samples were extracted with the optimized procedure. Schirmer's strip test in the absence of local anesthesia provided a standard, convenient, and non-invasive method for tear collection.

## Introduction

The precorneal tear film is comprehensively defined as a multi-layered structure consisting of three distinct layers: the outermost lipid layer, the middle aqueous layer, and the innermost hydrophilic mucin layer [1]. The superficial lipid layer is secreted by the Meibomian glands located in the tarsus and reduces evaporation of the aqueous layer. The middle aqueous layer is produced by the lacrimal glands, whereby the main lacrimal gland triggers reflex tears while the accessory lacrimal glands of Wolfring and Krause handle basal secretion. The mucin layer, primarily made up of goblet cells, contains essential glycoproteins that reduce the hydrophobicity of the epithelial cells [2]. Tears consist of four main types: (i) basal tears, for continuous ocular surface maintenance and deficiency linked to conditions like dry eye disease and Sjögren’s syndrome, (ii) reflex tears as responses to external stimuli, (iii) emotional tears associated with intense emotions, and (iv) closed-eyed tears generated during sleep to remove debris and maintain ocular homeostasis [2,3]. The human precorneal tear film, considered as vital as blood and urine, serves as both an optical refractive medium for clear vision and a protective barrier to moisturize the external ocular surface. Composed mainly of water, it is intricate due to the presence of proteins, electrolytes, lipids, carbohydrates, inorganic salts, and small metabolites, playing a crucial role in maintaining ocular health [4-8].

In recent years, tear fluid has emerged as a promising candidate for proteomic analysis. One of the most commonly used analytical methods for tear protein analysis is the combination of high-pressure liquid chromatography (HPLC) with mass spectrometry (LC-MS/MS) [4]. Label-free LC-MS/MS spectral-counting quantitative proteomics has been used to identify differential tear protein expression between dry eye and meibomian gland dysfunction pathologies [9]. In addition to this, the discovery of protease inhibitors, antibodies, complement proteins, and catalase has revealed that tear proteins play a critical role in regulating inflammatory processes, corneal wound healing, and protecting the eye from various pathogens and toxic oxidative compounds [10]. The protein content of tear fluid can vary depending on the condition of the ocular surface, providing valuable information about the health of the eye and the presence of infections [4]. The rich and diverse proteomic content of tear fluid also makes it a promising reservoir of biomarkers for the prognosis and diagnosis of diseases. Several potential biomarkers in tears were identified in ocular diseases such as keratoconus, dry eye disease, glaucoma, and ocular allergies [11]. The identification of biomarkers in tear fluid can provide valuable bioinformatic, genomic, and metabolic profiles that can be used to develop personalized medicine for effective therapy [4,6].

In routine and clinical trials, the collection of samples for evaluating a patient's health and pathology typically involves blood or urine collection rather than from the targeted cell or tissue. However, for ophthalmologists, the collection of tear samples is a safer and more convenient alternative to sampling from aqueous humor or vitreous humor, which is more invasive [8]. Compared to urine collection, tear collection can be easily carried out in a consultation room, which is particularly beneficial for patients with limited mobility [7]. Venipuncture is the most common invasive medical procedure for blood collection, and it is commonly done either from the inner forearm or the back of the hand. This procedure requires trained medical personnel to be carried out. Additionally, venipuncture has another concern including the risk of minor bruising and hematoma, which occurs in about 12.3% of patients [12]. While blood and urine samples are traditionally recognized as pathological indicators of systemic disease, there is growing interest in investigating tears as a potential source of biomarkers for detecting underlying systemic disorders. This is due to their non-invasive nature, less complex composition, and the advancements in proteomic assays [11]. Certain systemic diseases exhibit ocular manifestations, while others may not display any ocular complications. Dry eye syndrome is a common ocular complication that is linked to systemic diseases such as systemic sclerosis, cystic fibrosis, and diabetic retinopathy. However, tear fluid can now be explored as a non-invasive source of biomarkers for cancer and neurological diseases, even in the absence of ocular complications [11].

Although the tear film can be accessed easily and noninvasively, the collection of tear samples presents a significant challenge due to the minute amount of tear fluid and the instability of its components [6,7]. To address this challenge, there are two commonly used methods for collecting tear fluid: the Schirmer’s test strip and the microcapillary tube [4]. The Schirmer’s test strip is a paper test strip commonly used to measure the tear production of a patient for dry eye assessment. Microcapillary tubes are thin, narrow tubes that are gently inserted into the lower eyelid to collect tears by capillary action.

In this study, we aim to compare these two techniques of tear sample collection from various aspects including sample volume, tear protein composition, patient experience, and the technical aspect of sample collection. We also aim to evaluate the effect of topical anesthesia usage during the procedure and the choice of buffer for protein extraction.

## Materials and methods

This study was conducted using tear samples collected from eight randomized healthy volunteers, who did not have any ocular disease, from Hospital Sultan Abdul Aziz Shah (HSAAS), Universiti Putra Malaysia. The research protocols used in this study were approved by the Ethics Committee for Research Involving Human Subjects, which is the respective institutional review board of Universiti Putra Malaysia (UPM), Malaysia (Ref. no: JKEUPM-2021-1997).

Tear collection using Schirmer's test strip

Tear samples were collected from the volunteers, using Schirmer's test strips (Madhu Instruments Pvt. Ltd, New Delhi, India) with and without local anesthesia. Surface anesthesia was performed on the conjunctival sac by introducing one drop of 0.5% Alcaine eye drops (Alcon, Puurs, Belgium) containing 0.5% (w/v) proparacaine hydrochloride, five minutes before tear collection began [13,14]. The 5 mm end of Schirmer's test strip was folded and inserted into the lower conjunctival sac, and tears were allowed to diffuse into the strip until reached 20 mm on the strip scale [15]. During the sample collection process, respondents were allowed to blink freely [16]. Moreover, an additional strip with wetting of 10 mm and 30 mm length was also used for tear collection from respondents who were able to produce more tears. After tear collection, the Schirmer's test strip was cut into small pieces using sterile scissors. The tear-soaked strip was placed into either 300 µL of 1× phosphate-buffered saline (PBS) or 300 µL of 0.1 M ammonium bicarbonate (NH_4_HCO_3_). The microcentrifuge tubes containing the tears were kept at 4℃ before protein extraction.

Tear protein extraction from Schirmer's test strip

To extract tear proteins, the microcentrifuge tubes containing the cut-up strip were agitated over ice for two hours [15,17]. This was followed by centrifugation at 12,000 g for 10 minutes [5]. Centrifugation separated test strips to one side so that the soluble component could be collected. To quantify the concentration of tear proteins in PBS or NH_4_HCO_3_, the Pierce BCA Protein Assay Kit (23225; Thermo Fisher Scientific, Waltham, Massachusetts, United States) was employed and it utilizes a colorimetric reaction with bicinchoninic acid (BCA) to determine the protein amount. Following the manufacturer protocol in a microplate format, a nine-point calibration curve with concentrations ranging from 20 to 2000 µg/mL, was performed in duplicate using bovine serum albumin (BSA) as the standard (provided in the kit). Up to 25 µL of tear was used for the protein concentration measurement, which was performed in duplicate. The mixture was incubated for 30 minutes at 37℃, and the absorbance of eluted proteins was measured at 570 nm using an iMark Microplate Absorbance Reader (Bio-Rad Laboratories, Inc., Hercules, California, United States). The absorbance values from analyzed samples were interpolated from the standard curve equation to obtain the concentration of tear protein. Tear protein concentration was also quantified directly using a NanoPhotometer P300 (Implen, Munich, Germany). Finally, the tears were stored at −80℃ until further analysis.

Tear collection using microcapillary tube

To collect tears from the same group of eight volunteers, a drop of Rinz normal saline eye drops (Xepa-Soul Pattinson (Malaysia) Sdn Bhd, Melaka, Malaysia) was added to the lower cul-de-sac region to moisten the surface before a sterile 10 µL capillary tube was used to collect tear samples. The volume of capillary collected tears was around 10-20 µL since the tears obtained were not diluted with buffer or medium to prevent dilution [9,18]. However, since the minimum volume required for the Pierce BCA Protein Assay Kit is 50 µL for duplicates, the volume of tears collected using the microcapillary tube was insufficient to use with the assay kit. Therefore, the concentration of tear protein could only be measured directly by spectrophotometry (NanoPhotometer P300) at wavelength 280 nm. Tears were stored at −80℃ until further processing. At the end of the tear collection, subjects were asked for their preferred test material based on comfort.

Sodium dodecyl sulphate-polyacrylamide gel electrophoresis (SDS-PAGE)

To assess the quality and integrity of the protein profile, 10 µg of tear protein collected by Schirmer's test methods were separated by SDS-PAGE.

In the case of low tear concentrations, the volume required to achieve a protein load of 10 µg may exceed the maximum capacity of the well. Therefore, these samples were not at a set concentration but were loaded to the well's maximum capacity (20 μL). In contrast, for the microcapillary tube collection method, a total volume of 2 μL of tear was loaded into the well for analysis. The protein samples were denatured with 10% (w/v) β‑mercaptoethanol in 5× sample loading buffer and heated at 90℃ for five minutes. Once the 12% (w/v) resolving gel (0.1% (w/v) SDS, 1.5 M Tris-HCl, pH 8.6) was polymerized, the 5% (w/v) stacking gel (0.1% SDS, 0.5 M Tris-HCl, pH 6.8) was carefully layered on top of it and a comb was inserted into the gel to create sample wells [19]. All samples were loaded slowly and allowed to settle evenly at the bottom of the wells. A pre-stained molecular weight standard (3 μL) was also loaded into one of the lanes in the gel to serve as protein markers. Electrophoresis was performed using 1× Tris-glycine buffer (0.025 M Tris (pH 8.3), 0.192 M glycine, 1% (w/v) SDS) at a constant voltage at 90V for 10 minutes to allow migration of proteins from stacking gel to resolving gel, followed by 100 minutes at 100V for proteins separation at room temperature. The proteins in the gel were then stained with Coomassie Brilliant Blue R-250 (CBB R-250) for 30 minutes (0.1% (w/v) Coomassie blue R-250, 40% (v/v) methanol, 10% (v/v) acetic acid]. The stained gel was agitated gently in a destaining solution (10 % (v/v) methanol and 10 % (v/v) acetic acid) until the background became clear. The gel was then soaked in distilled water for long-term storage. The protein bands were visualized by eye.

Statistical analysis

Statistical analysis was performed using IBM SPSS Statistics for Windows, Version 29.0 (Released 2022; IBM Corp., Armonk, New York, United States) to assess the impact of various parameters (such as the use of anesthesia, quantitation method, and collection method) on the total concentration of collected tear proteins. For small sample sizes and skewed data, nonparametric tests such as the Mann-Whitney U test were performed for the two-group variables. Quantitative variables which are the mean total proteins were presented as mean ± standard deviation (SD). All statistical tests were two-sided with a significance level of 0.05. Therefore, a p-value of less than 0.05 was considered statistically significant.

## Results

Tear samples show multiple protein band profiles on gel electrophoresis

**Figure 1:**
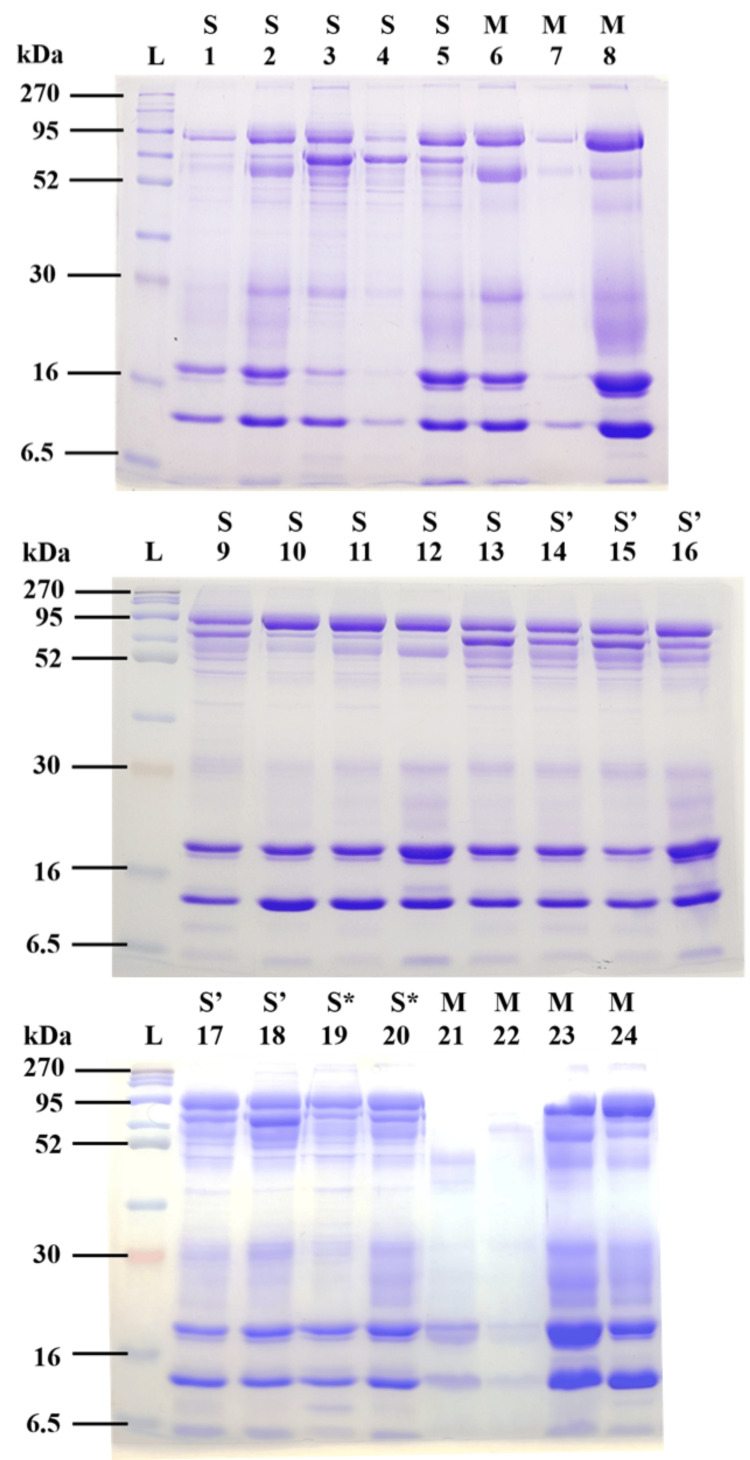
SDS-PAGE analysis of tear proteins collected using Schirmer's test strip and microcapillary tube. Lane L: BLUltra pre-stained standard protein marker (6.5-270 kDa) (GeneDireX, Taiwan); S: Tears collected using Schirmer's test strip without anesthesia in PBS (N=8); S’: Tears collected using Schirmer's test strip with anesthesia in PBS (N=5); S*: Tear collection using Schirmer's test strip without anesthesia in NH_4_HCO_3_ (N=2); M: Tears collected using microcapillary tube (N=7) Lane 1: 10 mm strip; Lane 4: 30 mm strip; Lane 2, 3, 5, 9-20: 20 mm strip; Lane 6-8, Lane 21-24: Microcapillary collected tear (N=7). PBS: phosphate-buffered saline; SDS-PAGE: sodium dodecyl sulphate-polyacrylamide gel electrophoresis

The SDS-PAGE protein separation profile of tear samples (N=24) from multiple collections of eight individuals using Schirmer's strip test and microcapillary tube are presented in Figure 1. The size of the tear proteins was determined using molecular weight standards concurrently electrophoresed with the protein samples. Tear samples showing similar protein band patterns suggest that the methods used for protein collection from tears are well-representative and robust.

Tear collection methods retrieved a high quantity of protein

The mean total protein from tear samples collected (without anesthesia) using the Schirmer's test strip (N=8) and microcapillary tube (N=7) are shown in Table 1. The mean protein concentration measured spectrophotometrically (NanoPhotometer P300) using the Schirmer's test strip method was 220.20 ± 67.43 µg compared to 87.42 ± 54.72 µg with the microcapillary tube method. Although the amount of protein collected was significantly different, both Schirmer's test strip and microcapillary tube methods yielded good tear protein concentrations. The Pierce BCA Protein Assay Kit was also used for Schirmer's test strip method and showed no significant difference from the spectrophotometric method. This measurement was not performed with the microcapillary tube as the original sample volume was insufficient.

**Table 1:** Mean total protein levels and corresponding discomfort level in tear samples collected using Schirmer's test strip and microcapillary tube. * Mann-Whitney U test with P=0.004, where P<0.05 taken as significantly different PBS: phosphate-buffered saline Manufacturer details: NanoPhotometer P300, Implen, Munich, Germany; Pierce BCA Protein Assay Kit, Thermo Fisher Scientific, Waltham, Massachusetts, United States

	Wetting length	N	Gender (F/M)	Buffer	Mean ± SD (µg)		Discomfort level
					NanoPhotometer P300*	Pierce BCA Protein Assay Kit	
Schirmer's test strip (Without anesthesia)	20 mm	8	7/1	PBS	220.20 ± 67.43	210.34 ± 59.46	Moderate discomfort, caused by the irritation of test strip
Microcapillary tube (Without anesthesia)	-	7	6/1	-	87.42 ± 54.72	-	No discomfort as it was handled by skilled staff

**Figure 2:**
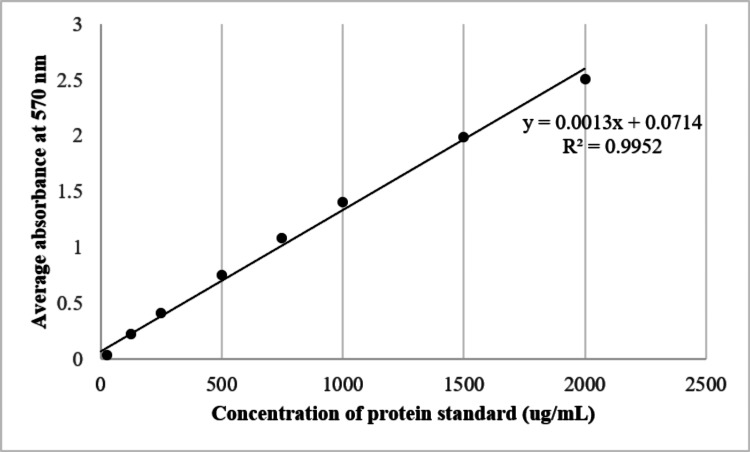
The dispersion of the standard curve and its equation for average absorbance obtained at 570 nm using Pierce BCA Protein Assay Kit for BSA. BSA: bovine serum albumin Manufacturer details: Pierce BCA Protein Assay Kit, Thermo Fisher Scientific, Waltham, Massachusetts, United States

The representative image of the protein standard curve set up using BSA standards is shown in Figure 2. The R^2^ value achieved was between 0.97 and 0.99. 

Optimum wetting length of Schirmer's test strip

The amount of protein obtained at wetting length of tear strips collected at 10 mm and 20 mm from sample 1 and another 20 mm and 30 mm strips from sample 2 are shown in Table 2. The Schirmer's test strip of length 20 mm was found to be optimal for tear collection, as it yielded the highest protein concentration compared to other strip lengths with less variation in tear protein concentration quantified by both protein measurement methods.

**Table 2:** Tear protein concentration from different wetting length using Schirmer’s test strip. All the tear samples were immersed in PBS and the concentration was estimated spectrophotometrically and using the protein assay. PBS: phosphate-buffered saline Manufacturer details: NanoPhotometer P300, Implen, Munich, Germany; Pierce BCA Protein Assay Kit, Thermo Fisher Scientific, Waltham, Massachusetts, United States

Schirmer’s test strip (Without anesthesia)				
Sample	Wetting length	Buffer	Total protein concentration (µg)	
			NanoPhotometer P300	Pierce BCA Protein Assay Kit
1	10 mm	PBS	96.9	58.2
	20 mm		261.3	223.8
2	20 mm	PBS	222.6	177.9
	30 mm		222.6	68.4

Standard buffers to elute tear proteins 

Tear samples from two respondents collected on two different Schirmer's test strips were used to compare the solubility of tear proteins in 1× (0.01M) PBS and 0.1M NH_4_HCO_3_. Both buffers were able to extract tear protein successfully and sufficiently. However, the mean total protein suspended in 0.1M NH_4_HCO_3_ was 140.25 ± 130.04 µg in NanoPhotometer and 200.25 ± 88.03 µg in BCA Protein Assay Kit, which was relatively lower compared to total tear proteins in PBS (Table 3). As seen in lane 19 and lane 20 of Figure 1, the tear proteins in NH_4_HCO_3_ had similar band profiles with that of PBS in SDS-PAGE despite having lower concentrations. Therefore, standard buffers such as PBS and NH_4_HCO_3_ were found to effectively elute sufficient tear protein.

**Table 3:** The mean total protein of tear samples collected using Schirmer’s test strip method in different buffers. PBS: phosphate-buffered saline; NH_4_HCO_3_: ammonium bicarbonate Manufacturer details: NanoPhotometer P300, Implen, Munich, Germany; Pierce BCA Protein Assay Kit, Thermo Fisher Scientific, Waltham, Massachusetts, United States

Schirmer's test strip (Without anesthesia)					
Wetting length	N	Gender (F/M)	Buffer	Mean ± SD (µg)	
				NanoPhotometer P300	Pierce BCA Protein Assay Kit
20 mm	8	7/1	PBS	220.20 ± 67.43	210.34 ± 59.46
20 mm	2	2/0	NH_4_HCO_3_	140.25 ± 130.04	200.25 ± 88.03

The use of topical anesthesia had no significant effect on the quantity of tear protein

The mean total protein from tear samples collected using the Schirmer's test strip (N=5) in the presence of anesthesia is shown in Table 4. These tear samples contain a large quantity of protein as measured by a nanophotometer (267.06 ± 76.78 µg) as well as a BCA protein assay (246.96 ± 85.11 µg). There was no significant difference in total protein obtained using both protein concentration measuring techniques for either group, indicating that they provide consistent quantification (p-value of 0.645 and 0.548, respectively). On the other hand, the use of anesthesia does not have a statistically significant effect on the total protein concentration since they have a p-value of 0.524 and 0.622, respectively (not shown in the table).

**Table 4:** The mean total protein of tear samples collected using Schirmer's test strip with/without anesthesia. PBS: phosphate-buffered saline Manufacturer details: NanoPhotometer P300, Implen, Munich, Germany; Pierce BCA Protein Assay Kit, Thermo Fisher Scientific, Waltham, Massachusetts, United States

Schirmer's test strip (Wetting length = 20 mm)						
Anesthesia	N	Gender (F/M)	Buffer	Mean ± SD (µg)		Mann-Whitney U test
				NanoPhotometer P300	Pierce BCA Protein Assay Kit	p-value
-	8	7/1	PBS	220.20 ± 67.43	210.34 ± 59.46	0.645
+	5	5/0	PBS	267.06 ± 76.78	246.96 ± 85.11	0.548

## Discussion

Tear sampling provides a promising alternative to other invasive techniques for the collection of biological samples in ophthalmology routine tests and clinical trials. Moreover, the collection of tear samples from patients is a safer and more convenient alternative to invasive procedures such as venipuncture. This can be easily carried out in a consultation room using a Schirmer's test strip, making it especially beneficial for patients with limited mobility or physical challenges [7]. The use of tear samples as a source of biomarkers enables clinicians to personalize therapy for patients with ocular diseases while monitoring the ocular surface and its response to treatments in a non-invasive manner [11,20].

There are several methods available for tears collection, including Schirmer's test strip, microcapillary tubes, cellulose acetate filter rods, polyester wick, and different ophthalmic sponges such as Merocel, Pro-ophta, and Weck-Cel that are made up of polyvinyl alcohol (PVA) and cellulose [6,14]. Each of these methods has its advantages and disadvantages, and the choice of method may depend on the specific research or clinical needs. Additionally, it is crucial for a tear collection method to be reliable and effective in obtaining a sufficient volume of tears for subsequent physical and chemical analysis as well as large-scale proteomic studies. Here, Schirmer's test strip and microcapillary tube were utilized as tear collection methods since it is the most cost-effective and straightforward approach [21].

SDS-PAGE protein separation profile of all tear samples exhibited a similar profile as demonstrated in previous studies [5,17,19,22,23]. According to Reitz et al. (1998), tear proteins can be separated into 31 protein bands by isoelectric focusing (IEF) in a pH gradient from 3 to 10 with hitherto unachieved resolution [24]. A total of 85% and 15% densitometric from these proteins were acidic and basic proteins, respectively [24]. Among the protein bands separated in SDS-PAGE in this study, several bands with higher intensity and consistently separated are believed to be lactoferrin (80 kDa), together with tear-specific pre-albumin, TSPA (18 kDa), and lysozyme (14 kDa) based on the molecular weight of the separated proteins [19,22-24]. In addition to the three proteins mentioned earlier, all samples in this study also exhibited the band of the protein G, which is approximately 30 kDa in size [19,23].

Although secretory immunoglobulin A (sIgA) was one of the main components in tear proteins, it was found to be poorly resolved by the 12% separating gel and sometimes the protein band was seen smearing at the upper position of the gel [23]. This might be due to the co-migration of sIgA with other unknown high molecular-weight proteins with similar molecular weight [21]. In the current study, the sIgA band was only distinctly visible at lanes 3, 5, 6, 8, 19, and 24 (Figure 1), perhaps a longer electrophoresis time would allow a better separation of protein bands. With additional steps, a notable difference in tear protein profile before and after reduction in SDS-PAGE becomes detectable [23]. For example, non-reduced sIgA appears as smear and diffuse bands, whereas after reduction by adding a reducing agent and boiling, sIgA is separated into two protein bands due to the dissociation of the heavy and light chain of sIgA. Simultaneously, the disappearance of protein G suggests that this protein is susceptible to reducing agents [23]. The decision to treat a protein with reducing agents before being analyzed with SDS-PAGE should be based on the specific protein being studied and the goals of the study [23]. Overall, the tear protein band profile using Schirmer's test strip appeared to be more standardized and consistent compared to that collected by the microcapillary tube.

The inconsistency of using microcapillary tubes has been demonstrated in this study. The intensity of the bands from tear samples collected with the microcapillary method as shown in lanes 7, 21, and 22 (Figure 1) was relatively lesser compared with other microcapillary collected samples. Despite that, one of the downsides of using Schirmer's test strip for tear collection is the potential for serum leakage caused by the mechanical stimulation of the paper on the conjunctiva. This may lead to elevated levels of serum-derived proteins such as human serum albumin and transferrin in the collected tear fluid when compared to that collected using the microcapillary method, as reported in previous studies [19,25]. Thus, the detection of serum albumin band on SDS-PAGE served as a rapid method for identifying serum leakage from the conjunctiva into the tears [23]. In the future, the intensity of protein bands stained with CBB R250 can be measured using a densitometric method by comparing the band intensity with a standard curve plotted using the densitometric volume against the concentrations of purified controls such as lactoferrin, human serum albumin, and lysozyme [19,24].

To determine the endpoint of tear collection, previous studies have employed various protocols. Some studies have allowed tears to migrate up to 20 mm on the Schirmer's test strip, while others have employed a five-minute duration after inserting the strip in the lower cul-de-sac region [5,7,15,17]. In this study, the endpoint of tear collection was decided based on a former method [15]. This is to ensure an adequate protein quantity is obtained for downstream analysis and to establish standardization as the protein amount is fixed at a specific length for all samples. Since the tear diffusion rate varies among individuals within a five-minute interval, therefore it becomes challenging to achieve standardization. Furthermore, we attempted to collect tears using Schirmer’s strips of 10 mm and 30 mm wetted lengths. It was found that tear collection using longer strips of 30 mm or more may not be feasible for every individual since the use of filter paper can be traumatic and cause discomfort in the conjunctiva of the eye [19,22]. The mechanical stimulation caused by the filter paper made it impractical for respondents to provide additional tear strips, particularly for variables such as different strip lengths and the use of NH_4_HCO_3_ buffer.

The short experiment here also concluded that 20 mm was the most effective wetting length. In general, longer wetting lengths are indicative of higher tear production, while shorter wetting lengths suggest insufficient tear production [22]. While there is a correlation between the length of strip wetness and total protein content, it may not be advisable to leave the Schirmer's test strip in the conjunctival fornix for an extended period as it could result in greater irritation and disruption of ocular surface homeostasis [26]. This may be the reason for the lower total protein obtained with the 30 mm wetting length. This difference may also be attributed to the reflex tearing dilution during the collection of tear samples for the 30 mm strip. Since the dilution factor could be associated with reflex tearing, the use of topical anesthesia was tested during sample collection. Primarily, the main purpose of the instillation of topical anesthesia was to reduce stimulation from the Schirmer's test strip and discomfort before tear collection [7]. This is because the increased volume of lacrimal gland secretion in a stimulated tear may contribute to the diluting effect of the tear [27,28]. In addition, a five-minute interval between the instillation of the anesthesia and the tear collection was to lessen the dilution due to the accumulation of anesthesia at the eye surface [21]. Based on participants' feedback, the topical anesthesia only provided numbness for the first five minutes, during which the eye feels nothing. However, after the initial five minutes, they begin to experience slight discomfort. In terms of protein content, although there were differences observed between the use of anesthesia, these differences were not found to be statistically significant. In summary, the use of anesthesia did not improve the tear collection process. Furthermore, anesthesia content may add to the contamination factor of the eluted tear proteins with external chemicals and affect downstream applications.

Although tear protein concentration was lower in the 30 mm strip when measured with BCA Protein Assay, spectrophotometer analysis showed no difference in protein concentration between the 20 mm and 30 mm strips. Similar findings were reported by Bocian et al. during their study on protein concentration measurement of snake venom using different quantification methods [29]. They discovered that the concentration of one of the venom proteins measured by four different methods (Pierce BCA Protein Assay Kit, 2-D Quant Kit (Cytiva, Marlborough, Massachusetts, United States), Bradford assay (Bio-Rad Laboratories, Inc.), and Qubit® Protein Assay Kit (Thermo Fisher Scientific Inc.)) was within a consistent range, whereas the readings from NanoDrop spectrophotometer (Thermo Fisher Scientific Inc.) were far outside this range [29]. Likewise, the concentration of major tear proteins such as lactoferrin, tear-specific pre-albumin, and lysozyme was found to fluctuate among different investigators in different studies. This inconsistency may be attributed to the different methodologies of protein quantifications employed, such as the electro-immunological method and scanning densitometry, as well as different tear sampling methods [19,25]. Thus, it is important to select the most appropriate method for measuring protein concentration, or at least a consistent method, as different quantitation methods may yield varying readings. In our studies, the differences observed in tear concentration measurement using two quantitation methods were not found to be statistically significant. This suggested that the two methods provide consistent measurements of tear concentration.

During the sample collection process, a single operator was responsible for inserting Schirmer's test strips to eliminate any variability in insertion technique between investigators [30]. The clinician in charge of the sample collection noted that collecting tears with a microcapillary tube was more challenging than using a test strip. This is because the tear in the lower conjunctival fornix does not get taken into the tube through capillary action. A similar situation was encountered by Ng et al., who reported that tear collection without stimulation resulted in an insufficient amount of tears [19]. To obtain a usable amount of tear samples, various methods were attempted to stimulate tear production, such as the insertion of filter paper, ingestion of hot Japanese horseradish, and irritation by ammonia vapor [19,25]. However, Ng et al. ultimately decided to use tears from the yawn reflex, as this method caused the least irritation and discomfort [19]. To address this issue in our study, we used saline eye drops (Rinz) to moisturize the ocular surface prior to tear collection, while taking care to avoid any external stimulation. However, this approach may introduce another challenge as the saline solution could potentially dilute the tear concentration.

In the current study, respondents reported their discomfort levels after experiencing both tear collection methods. The tear collection process using Schirmer's test strips was more unpleasant than using the microcapillary tube due to the prolonged duration that caused pain sensation, as this would require a longer time exceeding five minutes [16]. Moreover, different individuals required varying amounts of time for their tears to diffuse up to the desired length. Age factor and the potential relationship between age and tear secretion should also be taken into account [19]. Conversely, some respondents expressed concerns about using microcapillary tubes for tear collection as the eye is vulnerable and susceptible to injury, and they believed that the sharpness of the instrument could potentially cause harm. This situation was reported earlier in 2014 when healthcare workers who lacked research experience may have been hesitant to use a microcapillary tube, which involves inserting a pointed object into the eye [7]. Nevertheless, the use of a microcapillary tube is still a viable option as it can minimize the mechanical disruption of the ocular surface as compared to Schirmer's test strips [19,25]. As a result, we favored the use of the Schirmer's test strip over the microcapillary tube in this study, from the perspective of the operator and respondent. It is consistent with previous research findings that have shown Schirmer's test strips to be a widely acceptable and non-invasive method for tear collection in primary healthcare settings [7,20].

Downstream proteomic applications after tear collection include LC-MS/MS, enzyme-linked immunosorbent assay, and multiplex assay technologies such as Luminex (Thermo Fisher Scientific Inc.) [3,13]. The choice of NH_4_HCO_3_ as the buffer for dissolving tear proteins in LC-MS/MS is based on its compatibility with trypsin for protein digestion. If a non-compatible buffer is used, the tear solution may need to undergo purification and be resuspended with a specific buffer for digestion, which could result in a loss of proteins during the clean-up process. A study in 2023 of buffers such as PBS, sodium chloride, NP40, and ammonium bicarbonate, showed a combination of NH_4_HCO_3_ and NP40 generate higher protein yield [22]. This is due to the strong buffering capacity from NH_4_HCO_3_, which is further enhanced by a mild non-ionic surfactant, NP40. In the current study, however, tear protein concentration was highest in PBS compared to NH_4_HCO_3_ which contradicted the previous study mentioned above. There was also no significant difference comparing 0.1 M with 0.05 M NH_4_HCO_3_ in terms of tear protein elution based on the SDS-PAGE (data not shown). Therefore, NH_4_HCO_3_ can be used as a substitute for PBS as the preferred buffer for tear protein extraction [5,17,22,23]. Furthermore, longer incubation time of strips in a selected buffer has been shown to increase protein concentration compared to immediate elution [22]. As a result, it may be worthwhile to investigate the option of prolonging the incubation period in the buffer to enhance protein elution from the strips as well. However, it is important to consider that an extended incubation time may result in protein degradation, especially for proteins with a short half-life. Finally, it is crucial to select an appropriate method for measuring tear protein concentration due to the complex nature of tear proteomics [29].

Limitations of the study

The study's scope was constrained by its limited sample size. Augmenting the number of participants would strengthen the statistical significance. Only one sample was collected for each wetted length of 10 mm and 30 mm for the Schirmer's test strips due to the decline in tear production over time, which made it challenging to collect sufficient tears within the allocated time. Finally, the mechanical stimulation caused by Schirmer's test strips made it impractical for respondents to provide additional tear strips, particularly for variables such as different strip lengths and the use of NH_4_HCO_3_ buffer.

## Conclusions

The Schirmer's test strip is a better tear collection method compared to the microcapillary tube as it allows standardization of sample collection with higher tear protein level acquisition. The optimal wetting length of the Schirmer's test strip was 20 mm, and when eluted in 300 μL buffer, provided consistent volume for downstream applications. Topical anesthesia and different quantitation methods did not have any significant effect on tear protein concentration. Downstream application and analysis method of tear protein remains an important factor in determining the choice of tear sample collection method.
