# Correction: Tear Samples for Protein Extraction: Comparative Analysis of Schirmer's Test Strip and Microcapillary Tube Methods

**DOI:** 10.7759/cureus.c173

**Published:** 2024-05-10

**Authors:** May Ling Tham, Aidalina Mahmud, Maha Abdullah, Rafidah Md Saleh, Amirah Mohammad Razali, Yoke Kqueen Cheah, Niazlin Mohd Taib, Kok Lian Ho, Mazaya Mahmud, Muhammad Mohd Isa

**Affiliations:** 1 Department of Ophthalmology, Faculty of Medicine and Health Sciences, Universiti Putra Malaysia, Serdang, MYS; 2 Department of Community Health, Faculty of Medicine and Health Sciences, Universiti Putra Malaysia, Serdang, MYS; 3 Department of Pathology, Faculty of Medicine and Health Sciences, Universiti Putra Malaysia, Serdang, MYS; 4 Department of Biomedical Sciences, Faculty of Medicine and Health Sciences, Universiti Putra Malaysia, Serdang, MYS; 5 Department of Medical Microbiology and Parasitology, Faculty of Medicine and Health Sciences, Universiti Putra Malaysia, Serdang, MYS

This article has been corrected by the Editor-in-Chief to include the correct units of measurement as detailed below.

The fifth paragraph of the Materials and Methods section originally stated that: 

In the case of low tear concentrations, the volume required to achieve a protein load of 10 µg may exceed the maximum capacity of the well. Therefore, these samples were not at a set concentration but were loaded to the well's maximum capacity (20 mL). In contrast, for the microcapillary tube collection method, a total volume of 2 mL of tear was loaded into the well for analysis. The protein samples were denatured with 10% (w/v) β‑mercaptoethanol in 5× sample loading buffer and heated at 90℃ for five minutes. Once the 12% (w/v) resolving gel (0.1% (w/v) SDS, 1.5 M Tris-HCl, pH 8.6) was polymerized, the 5% (w/v) stacking gel (0.1% SDS, 0.5 M Tris-HCl, pH 6.8) was carefully layered on top of it and a comb was inserted into the gel to create sample wells [19]. All samples were loaded slowly and allowed to settle evenly at the bottom of the wells. A pre-stained molecular weight standard (3 mL) was also loaded into one of the lanes in the gel to serve as protein markers.

The units of measurement have been corrected to microliters as follows: 

In the case of low tear concentrations, the volume required to achieve a protein load of 10 µg may exceed the maximum capacity of the well. Therefore, these samples were not at a set concentration but were loaded to the well's maximum capacity (20 μL). In contrast, for the microcapillary tube collection method, a total volume of 2 μL of tear was loaded into the well for analysis. The protein samples were denatured with 10% (w/v) β‑mercaptoethanol in 5× sample loading buffer and heated at 90℃ for five minutes. Once the 12% (w/v) resolving gel (0.1% (w/v) SDS, 1.5 M Tris-HCl, pH 8.6) was polymerized, the 5% (w/v) stacking gel (0.1% SDS, 0.5 M Tris-HCl, pH 6.8) was carefully layered on top of it and a comb was inserted into the gel to create sample wells [19]. All samples were loaded slowly and allowed to settle evenly at the bottom of the wells. A pre-stained molecular weight standard (3 μL) was also loaded into one of the lanes in the gel to serve as protein markers.

The authors and journal deeply regret that this error was not identified and addressed prior to publication.

